# Supplementary material for: Associations between Maternal Dietary Patterns and Infant Birth Weight in the NISAMI Cohort: A Structural Equation Modeling Analysis
Source: Nutrients. 2021 Nov 12;13(11):4054. doi: 10.3390/nu13114054 (PMC8623182; doi:10.3390/nu13114054)
Supplement: Supplementary file 1 [file nutrients-13-04054-s001.zip › nutrients-1437696-supplementary.pdf]

Frame S1. Factors used in the estimation of daily consumption of foods included in the FFQ.

Reported Frequency	Daily Frequency
3 or more times/day	3*1=3
2 to 3 times/day	2.5*1= 2.5
1 time/day	1*1= 1
5 to 6 times/week	5.5/7= 0.79
2 to 4 times/week	3/7= 0.43
1 time/week	1/7= 0.14
1 to 3 times/month	2/30= 0.07
1 time/month	1/30= 0.03
Never/Almost never	0

Table S1: Food groups taken from the the FFQ and their respective foods. Santo Antônio de Jesus, Bahia, Brazil, 2012 a 2013.

Group	Pregnancy trimesters	Foods
**Cereals, roots and tubers**	First trimester<br>Third trimester	Rice; Pasta; Manioc Flour; Sweet Biscuits; Cakes; Savoury Biscuits; Corn/Maize; Oats; Couscous; Tapioca; Bread; Potato and Cassava.
**Legumes**	First trimester<br>Third trimester	Beans and Pidgeon Pea
**Vegetables**	First trimester<br>Third trimester	Lettuce; Chayote; Carrot; Collard Greens; Cabbage; Tomato; Pumpkin; Cucumber; Scarlet Eggplant; Onion; Garlic and Peppers
**Fruit**	First trimester<br>Third trimester	Banana; Papaya; Orange; Apple; Watermelon; Pineapple; Tangerine; Breadfruit; Avocado; Mango; Lime; Passion fruit; Guava; Natural Juice and Plantains.
**Sugars and sweets**	First trimester<br>Third trimester	Sugar; Caramels and sweets; Powdered chocolate and Guava paste
**Processed and industrialized foods**	First trimester<br>Third trimester	Ready-made products (Soft drinks; Artificial Juice; Stock; Ready-made Soup and Sauces). Cured meat products (Linguiça; Mortadella; Carne-do-sol; Jerky and Bacon).
**Milk and dairy products**	First trimester<br>Third trimester	Whole milk; Cheese; Full-fat Yoghurt and Requeijão.
**Meat and eggs**	First trimester<br>Third trimester	Eggs; Offal; Beef with bones; Boneless Beef; Chicken; Fish and Pork.
**Fats**	First trimester<br>Third trimester	Margerine and Butter
**Fried Snacks**	First trimester<br>Third trimester	Fried Snacks
**Coffee**	First trimester<br>Third trimester	Coffee.
